# The increasing cost of happiness

**DOI:** 10.1016/j.ssmph.2021.100949

**Published:** 2021-10-22

**Authors:** R.W. Morris, N. Kettlewell, N. Glozier

**Affiliations:** a Central Clinical School, Faculty of Medicine and Health, University of Sydney, NSW, Australia; b ARC Centre of Excellence for Children and Families Over the Life Course, Australia; c Economic Discipline Group, University of Technology Sydney, NSW, Australia; d Institute of Labor Economics (IZA), Bonn, Germany

**Keywords:** Subjective wellbeing, Household income, HILDA

## Abstract

A fundamental question for society is how much happiness does a dollar buy? The accepted view among economists and psychologists is that income has diminishing marginal returns on happiness: money and happiness increase together up to a point after which there is relatively little further gain. In this paper we estimate the relationship between income and subjective wellbeing over a 19-year period focusing on where the greatest change in the marginal return on income occurs and whether this change point has shifted over time. We formally test for the presence of a change point as well as temporal changes in the relationship between income and affective wellbeing (happiness), and income and cognitive wellbeing (life satisfaction), using household economic data from Australia between 2001 and 2019. The results indicate that the change point between affective wellbeing and income has increased over those 19 years faster than inflation (i.e., cost of living). This suggests that inequalities in income may be driving increasing *inequities* in happiness between the rich and the poor, with implications for health and recent government policy-goals to monitor and improve wellbeing.

## Highlights

• We investigate how the relationship between income and subjective wellbeing (happiness and life satisfaction) has changed over time.
• Detailed wellbeing data from Australia over an economically stable 19-year period provided an undisturbed view of the changes.
• Happiness was less responsive to income after a certain income level, and this level had almost doubled since 2001.
• Conversely the relationship between income and life-satisfaction had remained relatively constant over the same period.
• A steep association between happiness and income has become more prevalent this millennium, and likely to drive inequities in wellbeing between the rich and poor.

## 1 Introduction

A fundamental question for society is just how much wellbeing does a dollar buy? Increasing income is commonly associated with increasing happiness and subjective wellbeing, however a point at which subjective wellbeing no longer increases with income has also been widely observed (Clark et al., 2008; Dolan et al., 2008; Easterlin, 1974).

Subjective wellbeing is not a unitary entity (Diener et al., 2017). Psychology studies typically distinguish between life satisfaction: the cognitive appraisal of one's own accomplishments; and affective wellbeing: one's prevailing affective state, emotional mood, or everyday experience of happiness (e.g., Kettlewell et al., 2020). While measures of these distinct concepts are correlated, the distinction seems to be critical in understanding the impact of income on wellbeing (Howell & Howell, 2008). For instance, we have recently observed that a major financial gain substantially improved cognitive wellbeing for individuals without much impact on their affective wellbeing (Kettlewell et al., 2020). In a now classic paper, Kahneman and Deaton (2010) showed that affective wellbeing increased with log household income up to a point (USD75,000), but after that the slope between income and wellbeing was effectively zero: increasing income had no further effect (i.e., “income satiation”). Conversely cognitive wellbeing continued to increase with log income beyond USD75,000. Studies since then have confirmed that while both affective and cognitive wellbeing increase with income, (log) income is more strongly related to cognitive than affective wellbeing (Diener et al., 2013, 2017; Howell & Howell, 2008).

Fundamentally, any difference in slope between affective wellbeing (i.e., happiness) and income for the rich and poor represents an unacknowledged source of inequity (i.e., unfairness) in the distribution of wellbeing in the economy. While much commentary has focused on the zero slope at high income levels reported by Kahneman & Deaton, any steeper slope at lower income levels (relative to high income levels) means wellbeing is distributed more unequally among people with low incomes. For instance, the “satiety” point at USD75,000 in 2008 reported by Kahneman & Deaton was substantially more than the US median income of USD52,000 in the same year, indicating that the affective wellbeing of the poorest majority of the US population was tied to marginal changes in income while that of the richer minority was not.

While studies agree the marginal returns of income on affective wellbeing tend to diminish, the exact form of the functional relationship between affective wellbeing and income is contested: some studies show the presence of a zero-slope change point after which increasing income has no further effect (Jebb et al., 2018; Kahneman & Deaton, 2010); other studies have found the relationship is approximately log-linear with no discontinuity in slope (Killingsworth, 2021; Sacks et al., 2012; Twenge & Cooper, 2020). Rather than assume a log-linear relationship to characterise income and wellbeing, we formally test and compare different functional forms (linear, log-linear and piecewise-linear) to identify the point of greatest change in the marginal effect of income (including but not limited to a zero-slope change); and then determine whether that point is shifting over time. For instance, a right shift of the change point on the income distribution would represent an increase in the income level at which happiness is no longer tied to income, and thus indicate more inequality in wellbeing among the poor than the rich. Conversely, a decrease (left shift) would represent a more equal distribution of wellbeing across income levels.

Inequalities in the distribution of wellbeing are increasingly relevant to governments and policy-makers due to the growing recognition that increasing income does not necessarily lead to equal changes in wellbeing (Clark, 2018; Frijters et al., 2020). Even prior to COVID-19, the World Gallup Poll has observed that affective wellbeing has decreased over the past decade in western Europe, North America, Australia and New Zealand, despite increases in average income in the same countries (Sachs et al., 2019). However to date there has been little investigation of whether the relationship between income and affective wellbeing has changed over time, which may contribute to these trends. In particular, has the functional relationship between income and affective wellbeing, and therefore the distribution of happiness between rich and poor, become more or less equitable in the last few decades?

## 2 Methods

We used household economic panel data from Australia to provide the first investigation of whether changes in the functional form between income and wellbeing have shifted between 2001 and 2019. HILDA (the Household, Income, and Labour Dynamics in Australia survey) provides a representative sample of households in Australia with detailed measurements of income and subjective wellbeing each year. We distinguished between cognitive- and affective wellbeing as different components of subjective wellbeing, and evaluated how each varied with household income over time using linear, log-linear and piecewise-linear regression in each year.

### 2.1 Income

We used household after-tax income as the indicator of income and economic security (e.g., Kahneman & Deaton, 2010). The ‘real household annual disposable income’ was calculated from the self-reported combined income of all household members after receipt of government pensions and benefits and deduction of income taxes in the financial year ended 30th June of the year of the wave (e.g., 2001 in wave 1). This was then adjusted for inflation - the rise in the general price level of the economy - using the Australian Bureau of Statistics (ABS) Consumer Price Index, so that income in all waves is expressed in 2019 prices, to give real income.

The *equivalised* household income was obtained by adjusting for household size (the number of adult and child household members) using the ‘modified OECD’ scale (Hagenaars et al., 1994). Household income was divided by 1 for the first household member plus 0.5 for each other household member aged 15 or over, plus 0.3 for each child under 15. The equivalised income calculated for a household was then assigned to each member of the household.

### 2.2 Subjective wellbeing

Cognitive wellbeing was assessed by a single item question asked each survey: “All things considered, how satisfied are you with your life (0–10)”.

Affective wellbeing was determined by 9 questions in the SF-36 (9a to 9i). The SF-36 is a widely used self-completion measure of various aspects of physical, emotional and mental health (Ware, 2000), which has been validated in the Australian HILDA sample as a measure of health inequality (Butterworth & Crosier, 2004). A subset of 9 questions assess mental health and vitality, with four questions measuring agreement with positive aspects of mental health and vitality (i.e., “Feel full of life”, “Felt calm and peaceful”, “Have a lot of energy”, “Been happy”), and five questions measuring agreement with negative aspects (“Felt so down in the dumps nothing could cheer me up”, “Felt worn out”, “Been a nervous person”, “Felt down”, “Felt tired”). The response scale timeframe was the past four weeks and agreement was indicated on a six-point Likert scale. We have previously shown a summed score of these 9 questions distinguishes the impact of good and bad major life events in a bidirectional manner (Kettlewell et al., 2020). Supplementary analysis on the subset of positive and negative questions did not reveal any difference in the pattern of results between them (Supplementary materials, Fig. S5), and so we reverse scored negatively-phrased questions and calculated the sum of the nine questions so that higher scores represented better wellbeing. To aid interpretability, we rescaled the final sum to a score between 1 and 100, where 100 represents the maximum affective wellbeing achievable.

### 2.3 Modelling

We modelled the relationship between income and each wellbeing variable (cognitive and affective) using a linear model, log-linear model, and a piecewise-linear model with a single change point as a free parameter estimated from the data. The piecewise-linear model was chosen as the simplest extension of a linear model which can identify a change point (discontinuity in slope) in the relationship between wellbeing and income. For modelling, both measures of wellbeing and income were rescaled with a mean of zero and a SD of 1 (z-scores) for each year. As each year was modelled separately this does not affect the results, and the model parameter estimates were returned to their raw score units (e.g., real 2019 dollars) in figures and tables.

#### 2.3.1 Model design

We adopted a Bayesian approach for estimating the linear, log-linear and piecewise-linear model in the software Stan (Bürkner, 2017; Stan Development Team, 2019). The log-linear model was estimated after log transforming the income values, and the linear and log-linear model were then both estimated as:

(1) $$y_{i}∼N\left(\mu_{i},\sigma_{y}^{2}\right)$$

(2) $$\mu_{i}=\beta_{0}+X_{i}B$$

where $X_{i}$ was an individual's log household income (log($)) as well as other covariates (i.e., age, age^2^, sex, education, chronic illness, HILDA population weights; see below), and $y_{i}$ was an individual's wellbeing. Note that B is a column vector with a term 1 … p for each parameter in $X_{i}$.

The piecewise-linear model included a free parameter to represent the change point in income (ω) as well as the slope before the change point ($\beta_{1}$) and the slope after the change point ($\beta_{2}$):

(3) $$\begin{array}{c} \mu_{i}=\beta_{0}+\beta_{1}\left(x_{i}-\omega \right)\left(x_{i}\leq \omega \right)+\\ \beta_{2}\left(x_{i}-\omega \right)\left(x_{i}>\omega \right)+BX_{i} \end{array}$$

where $x_{i}$ was an individual's household income ($), and $X_{i}$ were the covariates-of-no-interest (see below for complete description).

The above models estimated population-level effects separately for each year (*t* = 2001 … 2019). Because we were interested in the location of the change point between income and wellbeing that existed across individuals within each year, we ignored the panel design of HILDA because the dependency between observations of the same person across years was orthogonal to our effects of interest.

The parameters of the piecewise-linear model used Gaussian priors centred at zero, with a regularized parameterization for the slopes and intercept of Normal(0, 0.1) for each *β*, and a slightly less skeptical regularization for the change point Normal(0, 0.5). These regularizing priors were selected to reduce overfitting while still allowing the model to learn the regular features of the sample, and so provide a more robust population estimate. They are skeptical priors and a prior predictive check confirmed they assume no relationship between income and wellbeing, with no difference in gradient before or after the change point (Supplementary materials, Fig. S6).

#### 2.3.2 Model selection

We compared the out-of-sample deviance of the log-linear and piecewise-linear model fits using the Widely Applicable Information Criterion (WAIC). The WAIC represents an approximation of the out-of-sample deviance that converges to the cross-validation accuracy in a large sample, with a penalty for the effective number of parameters (degrees of freedom). Thus using the out-of-sample deviance for model comparison in combination with regularizing priors in our model design is a dual strategy to reduce overfitting and penalize overfitting. As with other deviance metrics, smaller WAIC values are better (i.e., indicate more accuracy).

WAIC was defined as: WAIC = −2(lppd - *p*_WAIC_)

Where lppd (log pointwise predictive density) is the total across observations of the log of the average likelihood of each observation, and *p*_WAIC_ is the effective number of free parameters determined by the sum of the variance in log-likelihood for each observation (*i*).

#### 2.3.3 Parameter estimation

To determine the location of the change point (*ω*) between wellbeing and income, we modelled the relationship between income and wellbeing across individuals using the piecewise-linear model described above, and sampled the posterior probability of *ω* over 4000 interations. The complete posterior distribution of *ω* for each year is presented along with the median.

#### 2.3.4 Covariates

Covariates for age, age ^2^, sex, education, and chronic illness were included in each model above. Cross-sectional population weights for Australia provided by the University of Melbourne for each year were also included as a covariate to adjust for differences in the sample representativeness (by sex, broad age, marital status, region, and labour force status). Thus each model includes terms for the linear effect of each covariate, and age^2^ includes a term for the quadratic effect. Full-time students were removed, as well as individuals with an annual household disposable income that was negative or indicated as *topcoded* by the University of Melbourne. The results of sensitivity analyses to determine the effect of the covariates and exclusions on the change point parameters are included in Supplementary Materials (Figs. S2, S3, and S4).

## 3 Results

The broad demographic characteristics of the sample are presented in Supplementary Materials Table S1. Household income and average cognitive wellbeing levels increased between 2001 and 2019, while average affective wellbeing scores decreased slightly over the 19 years. The proportions of each sex and couples were stable over time, as were the average household size and SEIFA index. However age, education, and chronic health conditions tended to slightly increase over time. For instance, average age increased by 2.3 years over the 19 years of the survey, which is obviously less than would occur in a cohort study (Watson & Wooden, 2012). Changes in the workforce varied with economic circumstances.

### 3.1 Cognitive- and affective wellbeing have distinct relationships with income

**Fig. 1:**
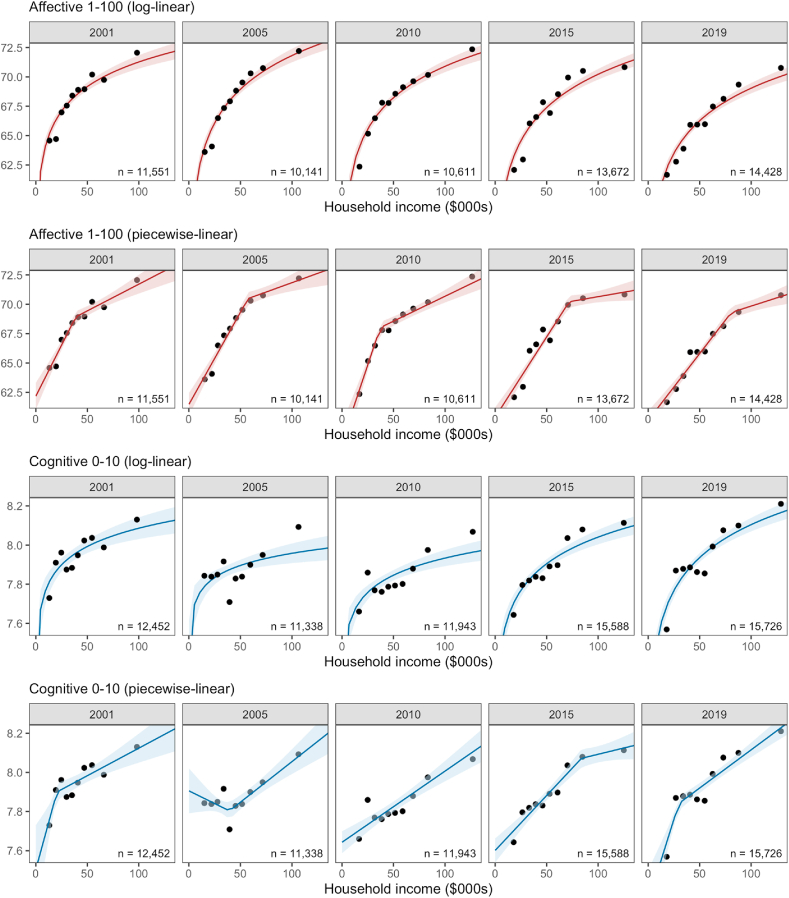
Association Between Household Income and Affective- (red) and Cognitive- (blue) Wellbeing from 5-years 2001–2019. The relationship between income and wellbeing across equal-sized income deciles, overlaid by regression lines from log-linear and piecewise-linear models (±95%CI). Wellbeing was measured as affective- (red) or cognitive- (blue) wellbeing. The total number of individuals contributing to each regression in each year are noted (n). . (For interpretation of the references to colour in this figure legend, the reader is referred to the Web version of this article.)

The relationship between household income and affective wellbeing (red) and satisfaction (blue) every four years is shown in Fig. 1. For each wellbeing variable we show the results of a log-linear fit (rows 1 and 3) and a piecewise-linear fit (rows 2 and 4). For visualization purposes only, due to the large number of individuals in each year, we display the mean levels of income and wellbeing for each (equal-sized) income decile, whereas the line-of-best-fit and 95% credible intervals (shaded) in each regression model are derived from *all individuals*.

The piecewise-linear relationship between affective wellbeing and income (Figure 1, 2nd row) was consistently and negatively inflected (affective wellbeing increased less with income after the change point). By contrast the piecewise relationship between cognitive wellbeing and income shown in the 4th row exhibited negative inflection (2001, 2015, 2019), positive inflection (2005), and no apparent inflection (2010).

The evidence from model selection revealed the piecewise-linear fit of *affective* wellbeing was superior to a log-linear or linear fit in each year with a penalised WAIC score credibly (95% interval) smaller than the log-linear fits for all but three of the nineteen years (and for all 19 years for the linear fits, see Supplementary Materials, Fig. S1). There was little evidence that any of the three models were reliably or credibly superior in representing the association between income and cognitive-wellbeing over the same period, albeit the linear model was better on average between 2002-2004 and 2007–2010 while the piecewise-linear model was better the remaining years. Overall the model comparison suggested that affective and cognitive wellbeing have distinct relationships with household income; affective wellbeing increases with income more rapidly at lower household income levels than higher income levels with a distinct change point in each year, while cognitive wellbeing tends to increase with income, most likely in a linear fashion. Others have also noted cognitive wellbeing (life satisfaction) has a stronger linear relationship with income than affective wellbeing and income (Howell & Howell, 2008), and so the results of our formal comparison provides some support for this.

### 3.2 Temporal trends in the association between income and affective wellbeing 2001–2019

**Fig. 2:**
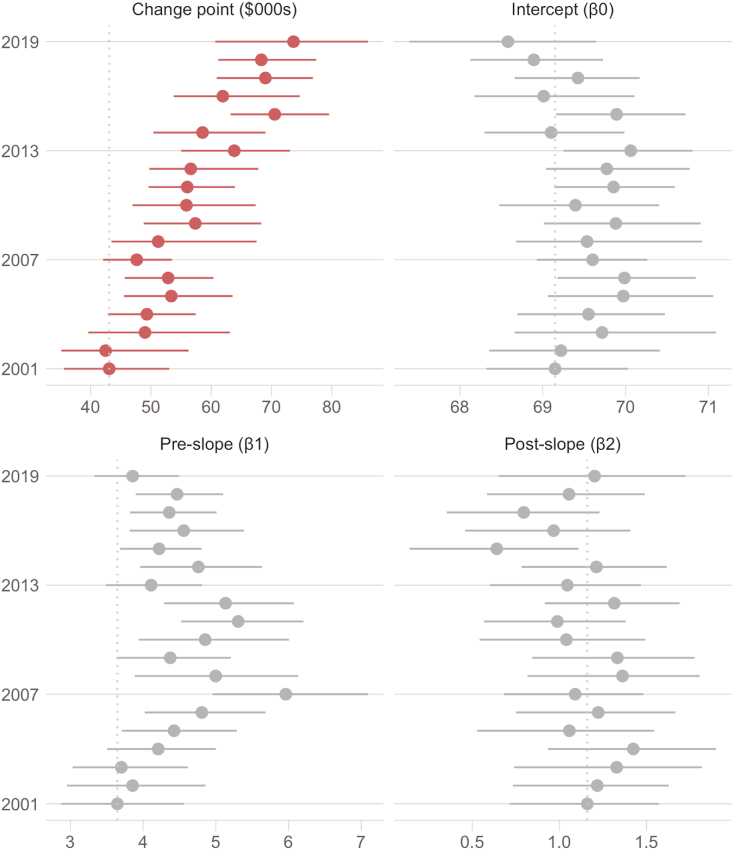
Posterior parameters of the affective-wellbeing ∼ income piecewise-linear model (real 2019 dollars). Posterior distributions of the change point parameter representing the location in real household income (real 2019 dollars), as well as the intercept, pre-slope and post-slope parameters in happiness units (0–100). Horizontal bar represents the 95% credible region and the solid point indicates the expected value (median) of each distribution. Vertical dotted line indicates the 2001 expected value (median) as a base year comparison.

Fig. 2 below presents the posterior distribution of each parameter from the piecewise-linear model regressing affective wellbeing on income: the change point (ω), the intercept (β_0_), the pre-change point slope (β_1_), and the post-change point slope (β_2_). Horizontal bars represent the 95% credible interval of the posterior distribution and so intervals which fall completely to the right of the vertical grey dotted line are credibly higher than the expected value of our base year, 2001.

The posterior estimates of the change point in the association between affective wellbeing and household income indicates that the probable location (i.e., the real income value) of the change point shows a systematic increasing trend since 2001. Changes to the other parameters of the function between income and affective wellbeing also occurred between 2001 and 2019 (i.e., the pre-slope, post-slope and intercept), but did not show any sustained trend over the period.

Both the pre-slope and post-slope parameters (β_1_ and β_2_) were credibly larger than zero in each year, indicating there was a reliable dependency between affective wellbeing and income at income levels both below and above the change point. However the pre-slope parameter was in general four to five times greater than the post-slope values, indicating that the relationship between affective wellbeing and income among people in lower income households was an order of magnitude stronger than those in high income households.

Changes in the other demographic variables (e.g., age, education levels and chronic illness) did not materially alter the trends just described. The effect of adding the covariates (age, age^2^, education, illness) on the piecewise-linear model parameters are compared in Supplementary materials (Fig. S2).

**Fig. 3:**
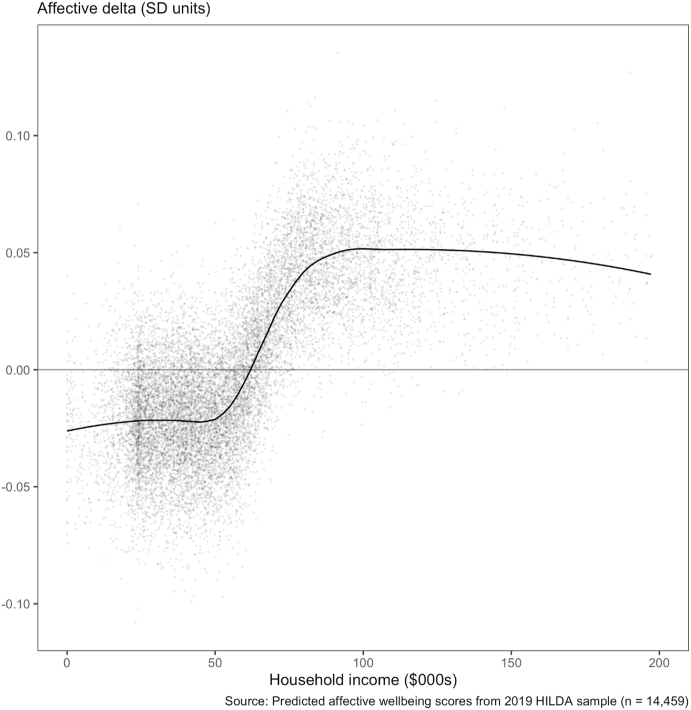
Counterfactual difference in affective wellbeing between 2019 and 2001 for a given level of income. The difference (Δ) in predicted affective wellbeing between 2019 and 2001 for the same n = 14,459 individuals. Values below zero on the y-axis indicate lower affective wellbeing predictions using the 2019 model compared to using the 2001 model. The smoothed overlay (solid line) indicates how the average affective wellbeing changes across the income distribution.

The change in parameter values between 2001 and 2019 indicates the relationship between affective wellbeing and income evolved over time. We determined the impact of this evolution on the distribution of affective wellbeing over the range of household income in 2019 in a counterfactual analysis. The counterfactual analysis is a hypothetical demonstration of how affective wellbeing would change if people in 2019 were subject to the function that existed in 2001, i.e., would affective wellbeing increase or decrease if the 2001 function was in place in 2019? This controls for changes in the sample which occur over time that are not related to affective wellbeing but could nevertheless contribute to changes in the distribution of affective wellbeing. For example, an increase in the range of (real) income levels in the economy between 2001 and 2019 could produce an increasing gap in affective wellbeing between the rich and poor - even with a stable relationship between income and affective wellbeing. Such changes in the sample characteristics may mask or confound the impact of the change point on the distribution of affective wellbeing without careful control. Because we were interested in the implications of the evolution of the function rather than changes in our sample characteristics *per se*, we estimated affective wellbeing levels for each person in 2019 (*n* = 14,459) using the 2001 function. These 2001 model-estimates were compared to (subtracted from) the 2019 model-estimates generated from the same sample (*n* = 14,459), to obtain the change (delta) in affective wellbeing for each person under the counterfactual. Thus, the delta is attributable to the evolution of the function over the 19 years. Fig. 3 below presents the 14,459 deltas from such a comparison, along with a smoothed mean (solid line) to summarize how the predicted difference in affective wellbeing is unequally distributed across the income range under the two models.

Fig. 3 shows that, on average, predicted affective wellbeing is higher for people with household incomes above $50 K under the 2019 function, when compared to the function from 2001. This is indicated by the average delta (solid line) falling above zero on the right side of the plot. Conversely, people with household incomes below $50 K, on average, had a decrease in predicted affective wellbeing under the 2019 function compared to 2001 function. These results are consistent with the picture in Fig. 1, which shows for example that affective wellbeing changed by −5.79% for people with income in the bottom decile between 2001 and 2019 compared to −2.92% for people in the top decile.

Of course the obtained deltas are due to changes in all the parameters of the function, including the slope before and after the change point. However because this comparison was performed on the same individuals from the 2019 survey, it held characteristics such as age, income, etc, constant that would otherwise be expected to change over time and possibly contribute to any difference in affective wellbeing distribution. In this way these results isolate the amount of change entirely due to the *evolution* of the function between 2001 and 2019, and demonstrates how this has contributed to a more unequal distribution of affective wellbeing between the rich and the poor over time in Australia.

### 3.3 The increasing cost of happiness in Australia

**Fig. 4:**
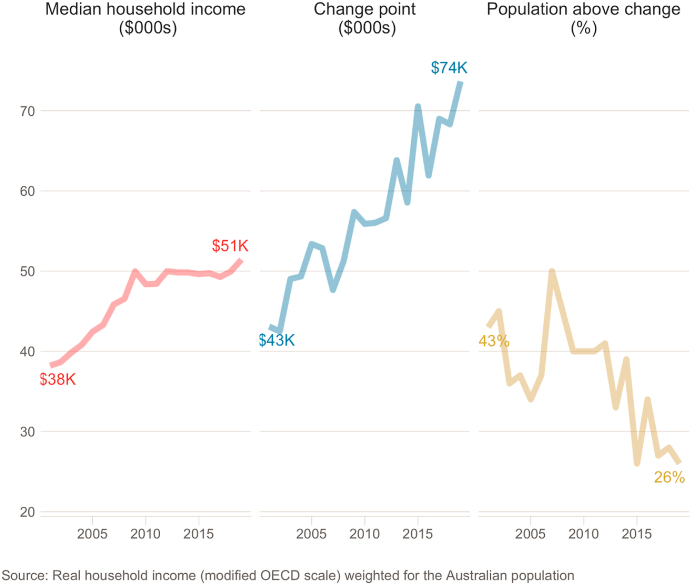
Rise in median income, change point, and population in Australia 2001–2019. Real household income has stagnated in Australia since 2009 (post GFC) while the change point between affective wellbeing and income has increased.

Any increase in the change point is likely to reduce the number of people who fall above it over time if this is greater than any increase in median income; i.e., over time a larger proportion of the population's affective wellbeing is responsive to marginal changes in their income than previously. Fig. 4 presents median household income levels weighted for the Australian population (by age, sex, marital status, labour force participation and geographical region). This shows the change point between income and affective wellbeing increased faster than rises in median household income between 2001 and 2019. The third panel shows that as a result, a smaller proportion of the Australian population in 2019 had a household income above the change point than in 2001.

## 4 Discussion

We found the marginal effect of household income on affective- and cognitive-wellbeing was positive but quite different: Affective wellbeing increased rapidly up to a point after which higher levels of income were associated with relatively less improvement, while cognitive wellbeing tended to increase linearly with income. Other studies have also reported that income has distinct effects on cognitive and affective wellbeing (Howell & Howell, 2008), and our results are consistent with that distinction. We also found a change point in the marginal returns of income on affective wellbeing in each year. Some influential studies have found a zero-slope change point in affective wellbeing after which increasing income had no further effect (Kahneman & Deaton, 2010). In our case we did not observe a zero-slope change point (“satiety” point), as the post-slope for each year was credibly above zero (Fig. 2); but the change point did represent a substantial decrease in dependency between income and affective wellbeing for those on higher income levels. Our novel finding was the change point increased faster than both inflation and median household income between 2001 and 2019. For the first time we have shown there has been a temporal shift in the change point between income and affective wellbeing over the 19 year period, such that affective wellbeing has become more dependent on income for more people - especially the poor and middle-class.

We refer to the change point after which increases in income no longer produce similar increases in affective wellbeing as the *cost of happiness*. After this point, affective wellbeing is no longer as dependent on household income, and the economic security it represents. Presumably after this point further increases in affective wellbeing depend more on other life factors (e.g., leisure time, social connections) than financial security. Cognitive wellbeing on the other hand appeared to show consistent increases with household income and we found no evidence of any change point. We have previously observed that cognitive- and affective-wellbeing appear to track distinct responses to financial gains and losses (Kettlewell et al., 2020), such that a major financial windfall produced less benefit to affective-wellbeing than cognitive-wellbeing whilst a major financial loss (e.g., bankruptcy) produced equal effects on both. The difference may reflect the importance of a numerical dollar value (e.g., bank balance, house value) when cognitively appraising one's life achievements, versus the relevance of that number to our everyday experience of joy and our prevailing mood.

An implication of the changing relationship between affective wellbeing and income is that income inequality may be driving increasing inequality in wellbeing. This is depicted in Fig. 3, where the difference in predicted affective wellbeing between 2001 and 2019 increased for incomes above $50K/year and decreased for incomes below that level (see also Fig. S8 in Supplementary Materials). Of course the counterfactual analysis represents the effects of temporal changes in all the model parameters not only the change point, but the change point was the only parameter which reliably changed over the 19 year period. For instance, Fig. 4 shows the change point represented a 9% increase over median income in 2001, while in 2019 it represented a 42% increase over median income. This increase relative to median income also represented a reduction from 43% to 26% in the proportion of people whose income fell above the change point. Thus we can see that over the last nineteen years the difference in income-related affective wellbeing between the rich and the poor has increased; while the affective wellbeing of an increasing proportion of people, including the middle-class, is more dependent on their financial security.

Other researchers have argued income has a satiety point on affective well-being, after which increasing income has no further effect (Jebb et al., 2018; Kahneman & Deaton, 2010). This is consistent with a change point after which the slope is zero. While we observed a change point, the slope afterwards was small but credibly larger than zero so our results are not strictly consistent with a “satiety” point. Our results emphasize the slope over income is not constant. Other researchers have also argued that affective wellbeing is a continuous log-linear function of income (Killingsworth, 2021; Sacks et al., 2012; Twenge & Cooper, 2020), which our results do not support and instead suggest there is a point (at least one) of discontinuity in the change of slope over income. We also show the change point has increased over time. Indeed Twenge & Cooper report the linear relationship between income and happiness has become steeper over time in the US General Social Survey (GSS). While we did not see any consistent trend over time in pre or post-slope changes in the piecewise-linear model of affective-wellbeing (e.g., Fig. 2), there was a credibly increasing slope in the linear model of cognitive-wellbeing with time (Table S3). It may be the single-item on general happiness in the GSS has more in common with the single item on life-satisfaction in HILDA than the more comprehensive 9-item, dual-valence questions on affective-wellbeing in the SF-36. Nevertheless, consistent with Twenge et al., our results imply that the difference in happiness between the rich and poor has been increasing over time (e.g., Figs. 6 and S8). Moreover our model reveals the income level around which the greatest difference appears.

### 4.1 Limitations of the present study

The amount of variance in wellbeing explained by income was small. While the amount of variance was small, the pre-change slope was reliably above zero and small changes in happiness over an entire population will aggregate. Moreover, financial gains and losses have one of the largest impacts on subjective wellbeing in within-subject/fixed-effect models (e.g., Kettlewell et al., 2020), and there are many reasons why cross-sectional estimates of the covariance would under-represent this (e.g., measurement error). Another limitation is that both types of wellbeing are measured on subjective scales, with different numbers of items for each. This would lead to differential measurement error, although *a priori* this would not cause the association between income and wellbeing to differ but rather reduce the precision of parameter estimates (which may explain our failure to distinguish the models of cognitive wellbeing). We also do not know whether small changes at different points on the scale represent larger changes in some other functional/real-world outcome (e.g., risk of suicide). At present, the real-world impact of changes in a subjective measure such as wellbeing is difficult to determine.

### 4.2 Conclusions

We justify a novel method, that challenges standard approaches, to show that the marginal effect of income on affective wellbeing is weaker for higher income groups (although not zero, i.e., “satiety”), and this point has been increasing over the past two decades. Australia has low levels of income disparity relative to many other OECD countries, and the Gini coefficient has changed little between 2001 and 2019 (APC, 2018), suggesting income inequality has remained steady over this time period. Our results do not conflict with this conclusion. Rather we suggest that even a static income distribution may have dynamic effects on happiness over time via changes in their joint relationship. This highlights the issue that while traditional measures of income and income inequality may be relatively stable and exhibit little change, their impact on wellbeing and health can still vary and change over time. As such, these results may well have relevance to other developed nations in North America and Europe which also enjoy stable levels of income inequality, but have stagnating incomes and declining happiness levels (Sachs et al., 2019). Establishing the links between income, wellbeing and health, and how inequalities in one drives inequities in the other, will be a critical aim as government begin to shift focus away from traditional measures of economic prosperity.

## Data statement

This paper uses unit record data from Household, Income and Labour Dynamics in Australia Survey HILDA conducted by the Australian Government Department of Social Services (DSS). All code and scripts used in the analysis are available online.

## Author statement

**RWMorris**: Methodology, Investigation, Validation, Formal Analysis, Data Curation, Writing- Original Draft, Visualization; **NKettlewell**: Methodology, Validation, Visualization, Writing- Review & Editing; **NGlozier**: Conceptualization, Resources, Writing- Review & Editing, Visualization, Supervision, Project Administration, Funding Acquisition.

## Funding sources

This research was supported by the 10.13039/501100015792Australian Government through the Australian Research Council's Centre of Excellence for Children and Families over the Life Course (Project ID CE2000100025). It uses unit record data from the Household, Income and Labour Dynamics in Australia (HILDA) survey. The HILDA project was initiated and funded by the Australian Government Department of Social Services (DSS).

## Ethics statement

This paper uses unit record data from the HILDA survey. The HILDA project is managed by the Melbourne Institute of Applied Economic and Social Research Melbourne Institute. The authors were not involved in the collection and dissemination of the data and as such did not seek ethics approval for this study, which only utilises pre-existing data.

## Declaration of competing interest

None.
