# Supplementary material for: The increasing cost of happiness
Source: SSM Popul Health. 2021 Oct 22;16:100949. doi: 10.1016/j.ssmph.2021.100949 (PMC8551651; doi:10.1016/j.ssmph.2021.100949)
Supplement: Multimedia component 1 [file mmc1.docx]

Supplementary material: The increasing cost of happiness

Draft: 19 October, 2021
Supplementary tables: 5
Supplementary figures: 8

**keywords:** Subjective wellbeing, household income, HILDA

## Supplementary Material

#### Sample characteristics

The broad demographic characteristics of the sample at each sixth year wave are presented below in Table S1. The linear trend as a function of time was estimated for each variable (linear regression of time for continuous variables and Cochrane-Armitage test in the case of binary variables), and Bon-Ferroni adjusted p-values for multiple comparisons are presented (k = 11).

##### Table S1. Sample characteristics

| Characteristic | 2001 N = 12,534^1^ | 2007 N = 11,385^1^ | 2013 N = 15,493^1^ | 2019 N = 15,790^1^ | p-value^2^ |
| --- | --- | --- | --- | --- | --- |
| Household income ($) | 42,553 (26,170) | 50,528 (45,267) | 55,863 (33,845) | 57,078 (34,078) | <0.001 |
| Life satisfaction | 7.9 (1.7) | 7.9 (1.5) | 7.9 (1.5) | 7.9 (1.5) | >0.9 |
| Happiness | 68.2 (16.8) | 68.4 (16.7) | 68.3 (16.8) | 66.2 (17.7) | <0.001 |
| Males | 5,949 (47%) | 5,348 (47%) | 7,363 (48%) | 7,491 (47%) | >0.9 |
| Age (years) | 46.0 (16.6) | 46.9 (17.4) | 47.4 (17.8) | 48.3 (18.3) | <0.001 |
| University Graduates | 2,303 (18%) | 2,539 (22%) | 4,012 (26%) | 4,568 (29%) | <0.001 |
| Unemployed | 474 (3.8%) | 278 (2.4%) | 568 (3.7%) | 528 (3.3%) | >0.9 |
| Couples | 8,682 (69%) | 7,721 (68%) | 10,564 (68%) | 10,686 (68%) | 0.14 |
| Chronic illness | 3,128 (25%) | 3,315 (29%) | 4,974 (32%) | 4,865 (31%) | <0.001 |
| SEIFA | 5.5 (2.9) | 5.6 (2.8) | 5.6 (2.8) | 5.6 (2.8) | >0.9 |
| Household size | 2.9 (1.5) | 2.8 (1.4) | 2.8 (1.5) | 2.7 (1.4) | <0.001 |
| ^1^Mean (SD); n (%) | | | | | |
| ^2^linear trend analysis (parametric); Cochrane-Armitage test | | | | | |

Household income and average life satisfaction increased between 2001-2019, while average affective wellbeing score decreased slightly over the 19 years. The proportions of each sex were stable over time, as was the proportion of people living as a couple (Couples), average household size and SEIFA index. The percentage unemployed varied with economic conditions however there was no significant positive or negative linear trend. Age and chronic health conditions tended to increase over time. The slight increase in the age of the panel over time (2.3 years) was substantially less than that of a cohort study which would be 18 years. Education levels (i.e., percentage of university graduates) also tended to increase over time.

Generally, Table S1 supports the view that the HILDA sample was a relatively stable representation of Australian socioeconomic conditions between 2001 and 2019. The importance of the specific demographic variables with a significant linear trend (age, age^2^, education, illness) were tested in regression models of affective wellbeing as covariates (see below Figure S2).

#### Model Comparisons

We compared the out-of-sample evidence for the piecewise-linear models with the log-linear and linear models of income on wellbeing in each year, using the difference in WAIC deviance scores (i.e., WAIC_piecewise_ — WAIC_log_, WAIC_piecewise_ — WAIC_linear_). Since smaller deviance scores indicate better fit, a difference in deviance scores less than zero (i.e., negative) indicated evidence for the piecewise-linear model; a difference greater than zero (i.e., positive) indicated evidence for the other model (log-linear or linear).

##### Figure S1. Model comparisons (WAIC_piecewise_ vs WAIC_linear_, WAIC_log_)


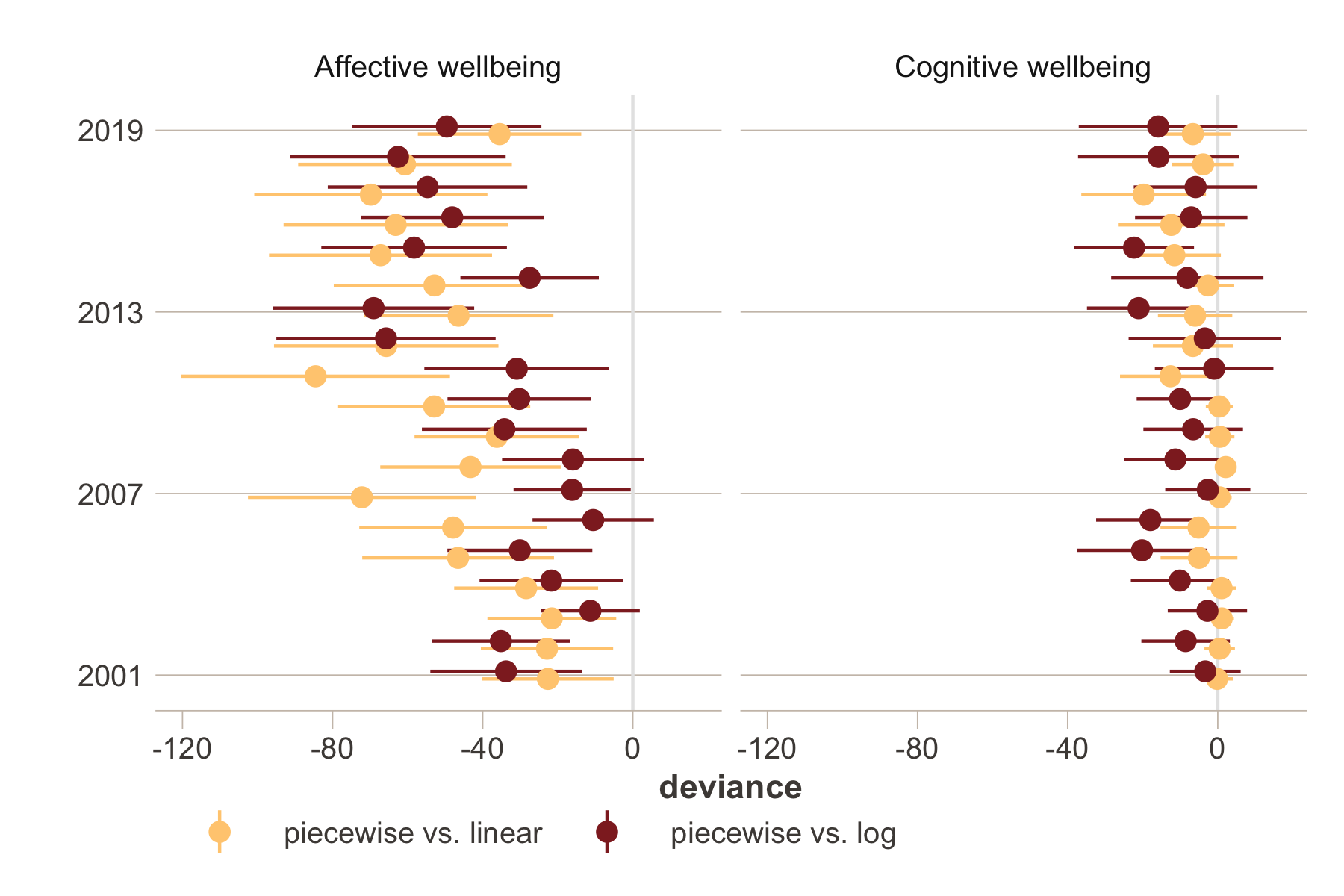


Figure legend: Differences in WAIC scores (deviance units) for a piecewise-linear fit over a log-linear fit (red) or over a linear fit (yellow). The filled circle indicates the mean of the difference and the horizontal bars represents the 95% interval. A difference below zero (grey vertical line) represents support for the piecewise model, and a difference above zero is support for the other model.

The difference in WAIC scores for each model revealed the piecewise-linear fits of affective wellbeing, but not cognitive wellbeing, provided superior out-of-sample accuracy over a log-linear or linear fit in each year. Figure S1 shows the differences in WAIC scores clearly distinguished the advantage of the piecewise-linear fits for affective wellbeing with most 95% intervals entirely below zero for each year (exceptions occurred for the piecewise vs. log fit in 2004, 2006 and 2008, where the piecewise fit was still superior on average). By contrast, the difference in WAIC scores for the cognitive wellbeing models was not as clearly distinguished from zero for most years, since the 95% intervals of the WAIC differences with either the log-linear fit or the linear fit overlapped zero in most years. Indeed in the years between 2002 to 2010 the linear fit was superior to the piecewise-linear fit on average, with the mean WAIC difference above zero (positive). Overall the evidence suggested that affective wellbeing has a piecewise relationship with household income; implying the existence of a change point or difference in slopes at different levels of household income. Evidence of the effect of income on cognitive wellbeing, on the other hand, was more equivocal, albeit with some indication of a linear relationship in the early part of the 21st C.

**Conditional vs unconditional models of affective wellbeing**. Table S1 (above) showed the specific variables that changed over the 19 years between 2001-2019. In particular, the number of university graduates, average age and the incidence of chronic illness increased slighty over the period. Including these variables as covariates in any model (i.e,. a conditional model such that *P*( $y$ | income, age, education illness)) will improve the in-sample fit (e.g., R^2^), and also adjust the parameter estimates of interest (e.g., change point) conditional upon their common association with affective wellbeing. Given the general aim of the main paper was to describe the temporal change in functional form between affective wellbeing and income in Australia, regardless of any other variable (i.e., an unconditional model of *P*($y$| income)) or sampling differences, neither the in-sample fit nor the conditional model estimates are directly relevant. Nevertheless, the importance of these variables in mediating the changes we observed in the unconditional model are relevant, and so we calculated the estimates from conditional piecewise models including age, age^2^, sex, education (university degree), chronic illness for each year below to confirm the pattern of temporal change was similar. Figure S2 shows the addition of these covariates did not change the trend in change point parameter estimates (red) relative to the unconditional parameter estimates (grey) over the 19 years.

#### Sensitivity tests of model parameters

##### Figure S2. Conditional model parameters


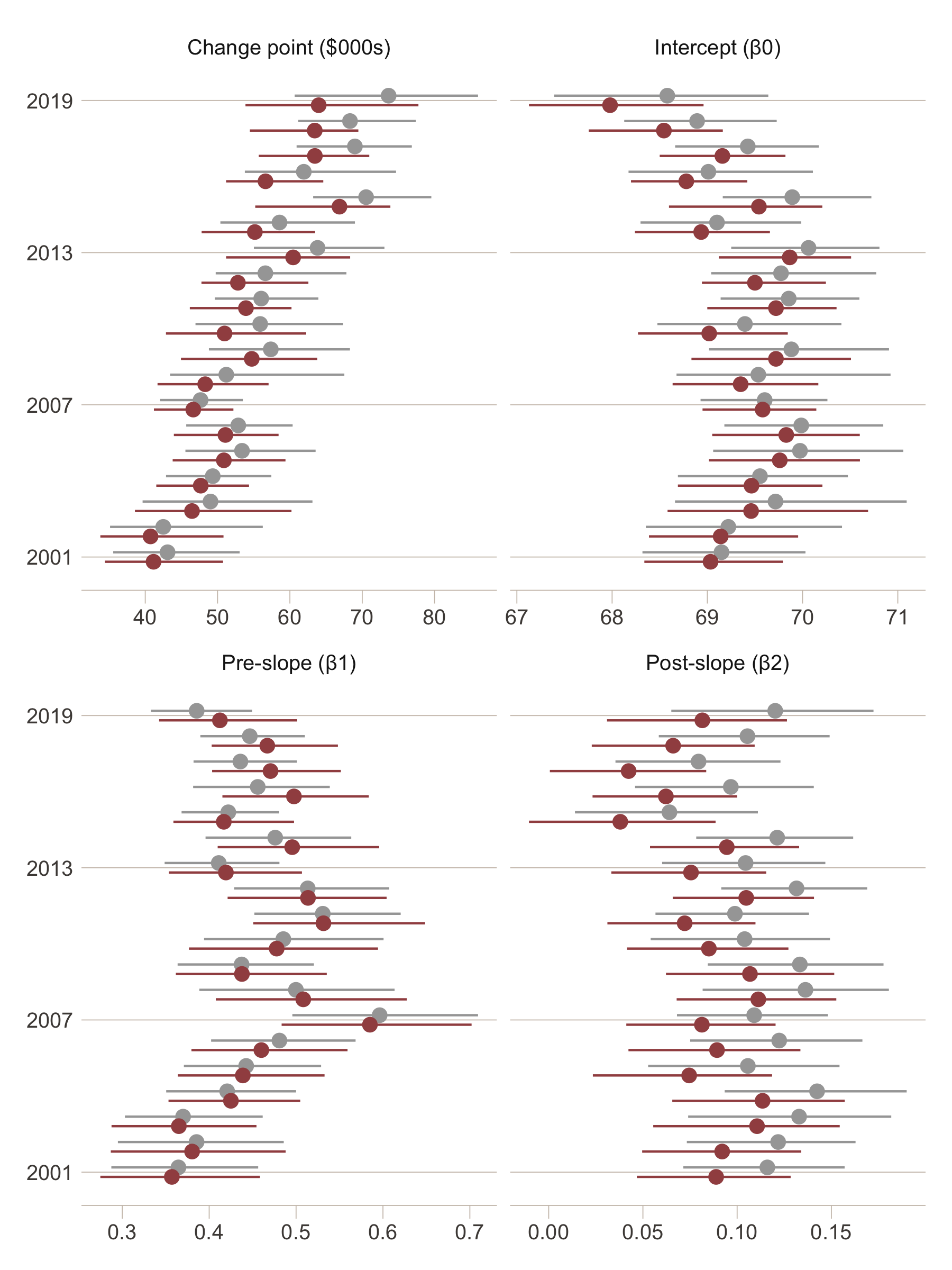


Figure legend: Comparison of the conditional parameter estimates (red) and unconditional parameter estimates (grey) in the piecewise-linear model of income on affective wellbeing. The conditional models included covariates for age, age squared, sex, education, and illness. Horizontal bar represents the 95% credible region and the solid point indicates the expected value (median) of each distribution.

The parameter estimates of the conditional model (red) were adjusted for the broad demographic variables which tended to increase over the 19 years in our sample (age, education, illness); however the pattern of changes over time was comparable to the unconditional model (grey), indicating these temporal trends were not responsible for the evolution in the change point between income and affective wellbeing.

The unconditional parameter values are also presented in Table S2 below (Parameter and fit summary statistics).

**Including income topcodes in models of affective wellbeing**.

Household income values for high income individuals are substituted with the average income value of all those above a certain threshold (topcoded). This is performed by the University of Melbourne to preserve anonymity, and we excluded people marked as topcoded in the main analysis (highest *n* in any year was *n* = 103 in 2019). Here we present the parameter estimates of the affective wellbeing models with topcodes included, and compare them with parameters from models which exclude them (as in the main text).

##### Figure S3. Model parameters including topcoded individuals


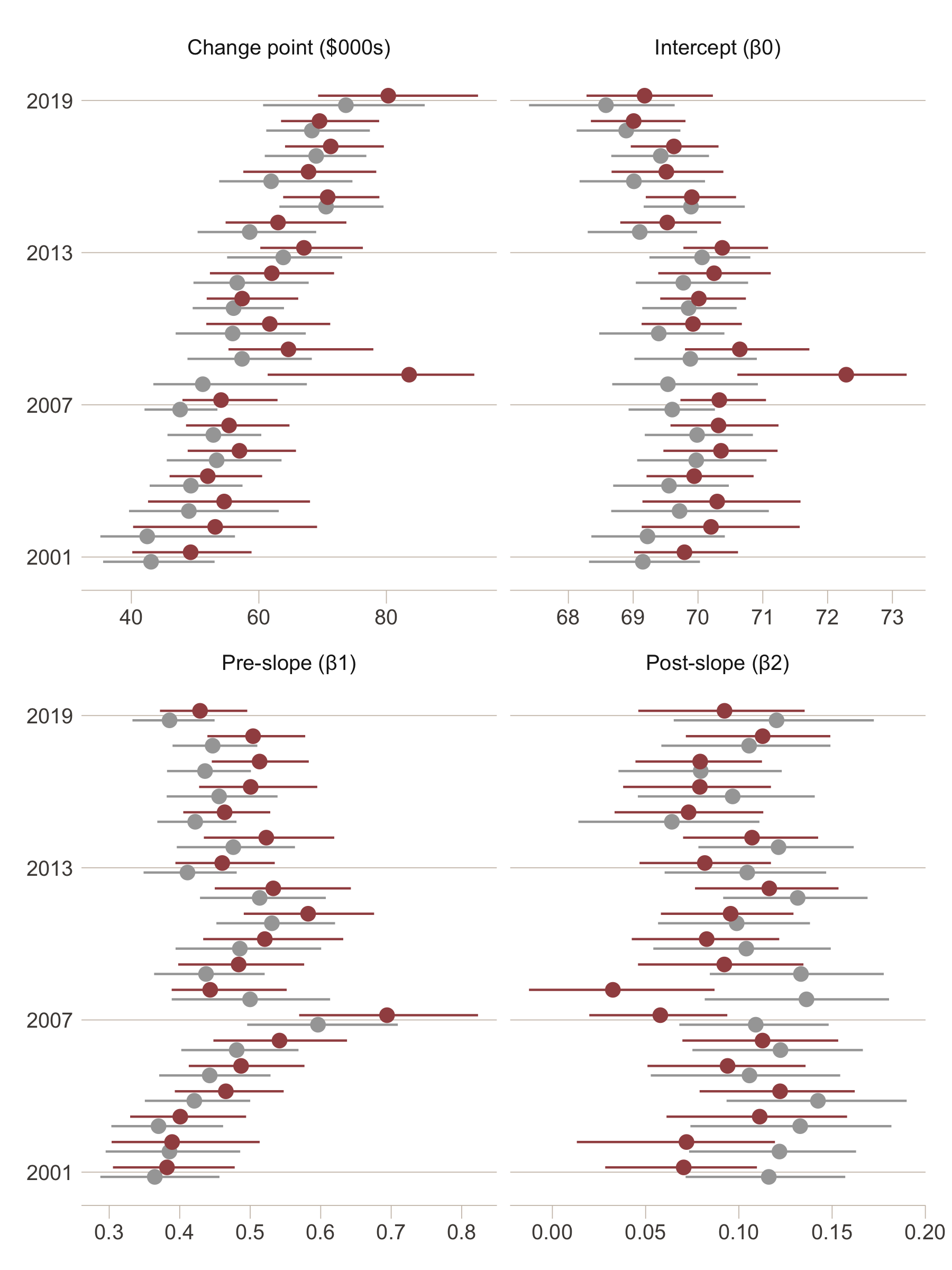


Figure legend: Comparison of the topcoded parameter estimates (red) and parameter estimates excluding topcodes (grey) in the piecewise-linear model of income on affective wellbeing. The topcoded models included n = 26 to 130 extra (high income) individuals in each year. Horizontal bar represents the 95% credible region and the solid point indicates the expected value (median) of each distribution.

Included topcoded individuals adds some variation in the parameter estimates (e.g., 2008), but did not change the overall pattern observed over time (e.g., the changepoint continued to increase over time).

**Unadjusted household income models of affective wellbeing**.

Household income values were adjusted for household size in the main analysis. The *maximum* household sizes varied from 10 (2017, 2019) to as high as 17 (2016), and so adjustment accounts for larger households needing more resources to achieve the same standard of living as a smaller household, and for some ‘economies of scale’ when sharing living costs. For example, two people living alone in two separate households typically incur higher living costs in total than two people living in one household. Here we present the parameter estimates of the affective wellbeing models without adjustment for household size, and with adjustment (as in the main text).

##### Figure S4. Model parameters unadjusted for household size


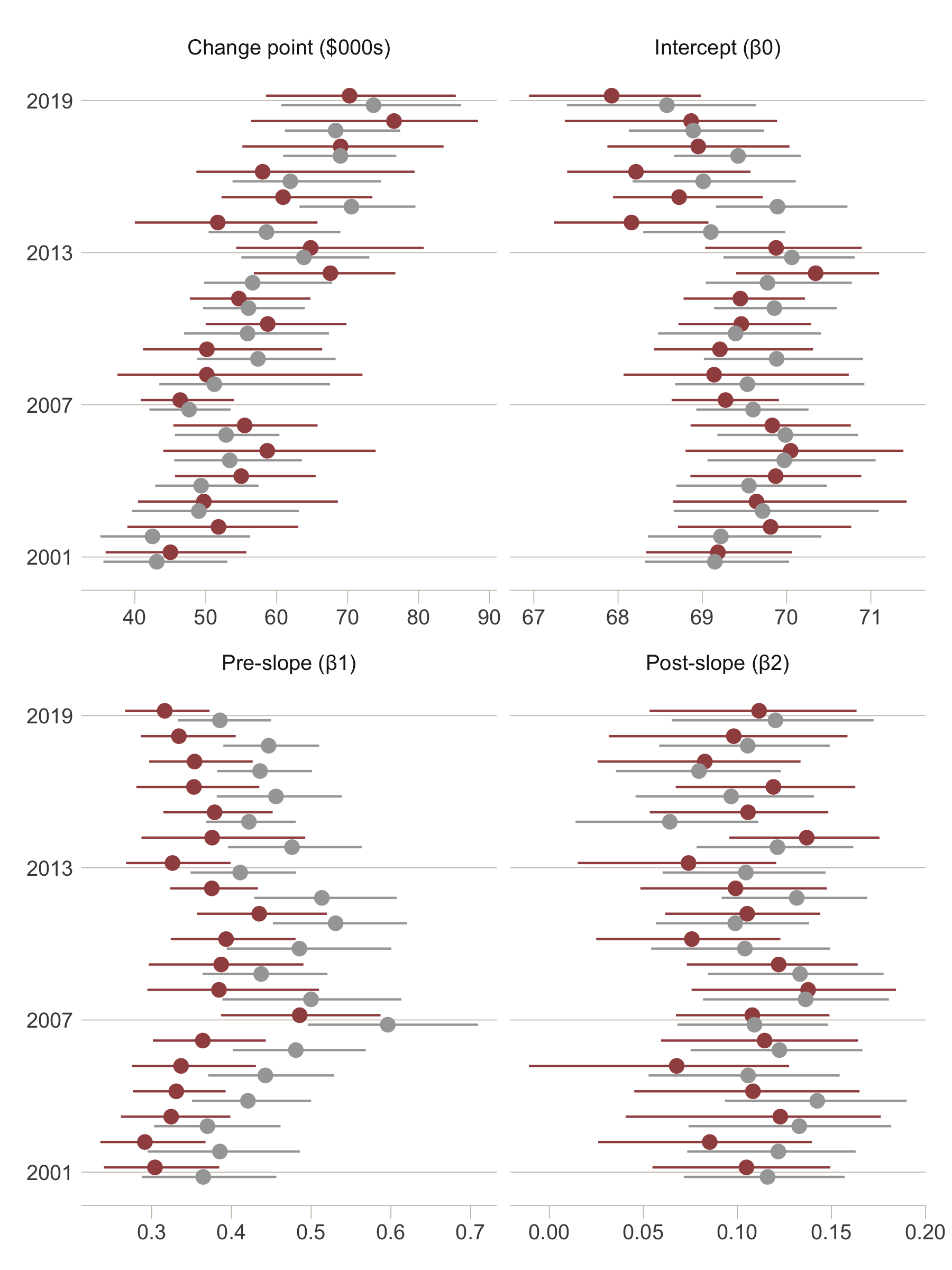


Figure legend: Comparison of the parameter estimates unadjusted for household size (red) and parameter estimates adjusted for size (grey) in the piecewise-linear model of income on affective wellbeing. Horizontal bar represents the 95% credible region and the solid point indicates the expected value (median) of each distribution.

Omitting the adjustment for household size on household income appears to reduce the $\beta_{1}$ parameters in each year, but there is little systematic effect on the other parameters and the changepoint continues to increase over time.

**Positive and negative components of affective wellbeing**. Affective wellbeing includes both positive and negative components, and both these components are assessed by multiple quesstions in the SF-36 item 9. To determine whether the increasing change-point in income on affective wellbeing we observed was due to positive or negative affect, we fit a piecewise-linear model of income on positive and negative affect separately. The four questions dealing with positive affect (i.e., “Feel full of life”, “Felt calm and peaceful”, “Have a lot of energy”, “Been happy”) and the five negative affect questions (“Felt so down in the dumps nothing could cheer me up”, “Felt worn out”, “Been a nervous person”, “Felt down”, “Felt tired”) were summed together separately to create distinct scores for positive and negative affect. The negative affect score was reversed so that higher scoes indicated less negative affect. Household income was then regressed against each score using the piecewise linear model (Equation 3), as described for the overall affective wellbeing score in the main text. Figure S5 below shows that the change-point in income increased over the period 2001-2019 for both positive (pink) and negative (blue) affect, indicating a similar trend in both components of affective wellbeing over the 19 years.

##### Figure S5. Positive and negative affect parameters


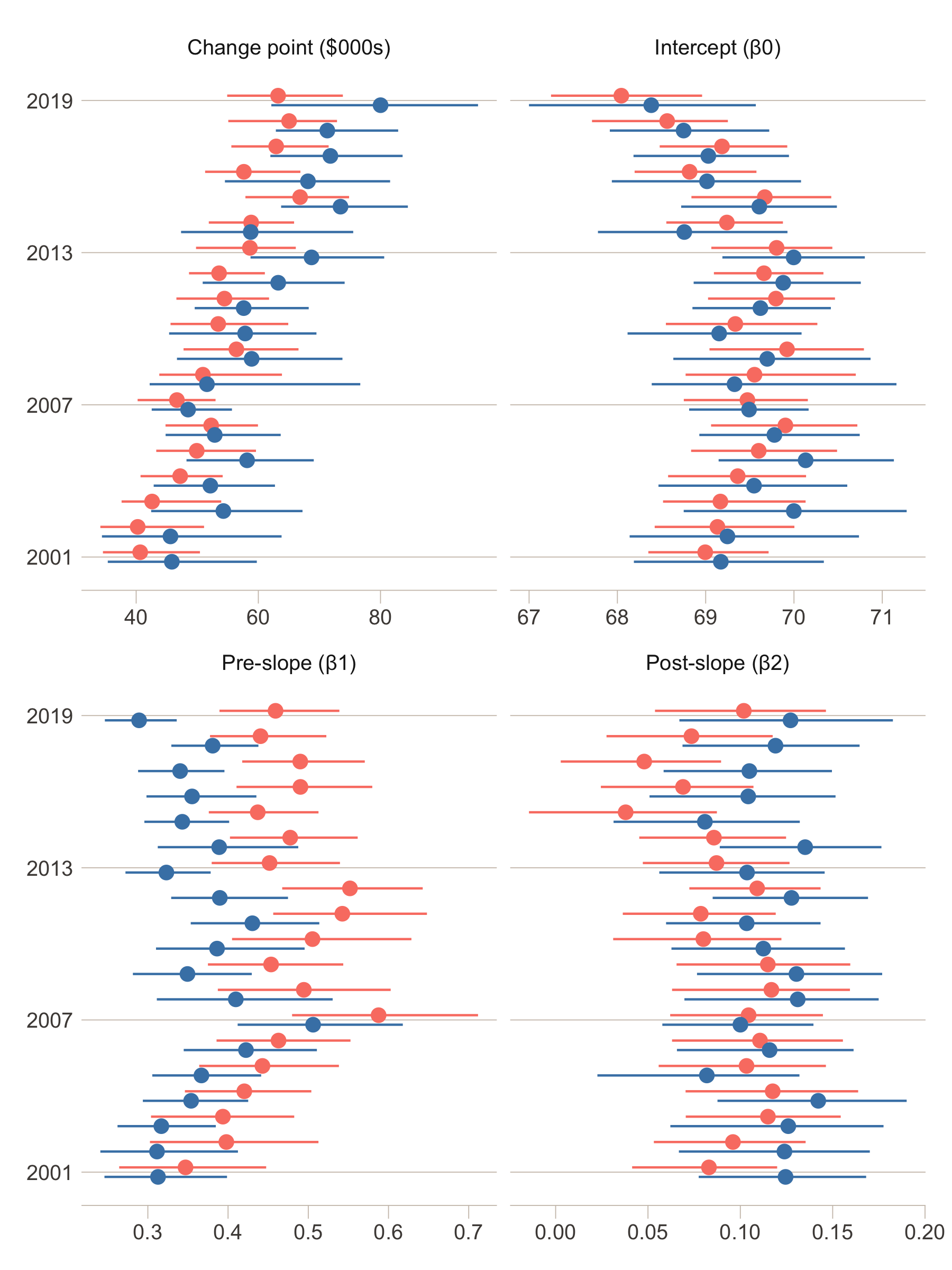


Figure legend: Comparison of the parameter estimates from positive items (pink) and negative items (blue) in the piecewise-linear model of income on affective wellbeing. The positive model regressed income against a sum of positive affect questions in the SF-36, while the negative model regressed income against a sum of negative affect questions (reversed scaled to match the positive affect score). Horizontal bar represents the 95% credible region and the solid point indicates the expected value (median) of each distribution.

Regressing income against the positive and negative components of affective wellbeing separately did not reveal substantially different trends for each. Both showed an increase in change-point income value over the 19 years, which by 2019 was credibly different from the base values in 2001. Changes in the other slope and intercept parameters were either not substantial or not credibly different from the 2001 values according to the 95% credible intervals.

#### Sampling and fit statistics

Below we provide sampling statistics (e.g., $\hat{R}$), as well as prior predictions and residual plots.

Model fitting was performed in RStan (v2.21.2) using the brms (v2.14.4) package (Bürkner, 2017; Stan Development Team, 2019). There were *N* = 59,876 total observations. For each Bayesian model presented in this report, 4000 samples (post-warmup) were drawn using sampling(NUTS).

For all models estimated with RStan presented in this report we confirmed the absence of divergent transitions; the $\hat{R}$ for each parameter approached 1 indicating convergance; and the effective sample size (ESS) for each parameter was *n* > 300, which is a conservative minimum threshold for estimation.

Table S2 below shows the sampling statistics for each piecewise-linear model fit of affective wellbeing and income in each year.

##### Table S2. Sampling statistics for piecewise-linear fit of affective wellbeing and income

|  | | | Bulk ESS |  | | | Tail ESS |  |
| --- | --- | --- | --- | --- | --- | --- | --- | --- |
| Year | β0 | β1 | β2 | ω | β0 | β1 | β2 | ω |
| 2001 | 1,488 | 1,852 | 1,894 | 1,366 | 2,151 | 2,441 | 1,971 | 2,009 |
| 2002 | 1,116 | 1,426 | 1,601 | 1,041 | 1,151 | 1,984 | 1,560 | 1,080 |
| 2003 | 1,164 | 1,653 | 1,536 | 1,092 | 1,253 | 1,506 | 1,365 | 1,261 |
| 2004 | 1,797 | 1,997 | 2,061 | 1,722 | 2,090 | 2,020 | 2,106 | 2,019 |
| 2005 | 1,255 | 1,730 | 1,669 | 1,150 | 1,522 | 2,034 | 1,776 | 1,705 |
| 2006 | 1,370 | 2,049 | 1,630 | 1,389 | 1,586 | 1,865 | 1,660 | 1,362 |
| 2007 | 1,491 | 1,890 | 1,833 | 1,460 | 1,963 | 2,045 | 2,206 | 1,488 |
| 2008 | 1,005 | 1,160 | 1,281 | 960 | 502 | 770 | 838 | 471 |
| 2009 | 1,350 | 2,008 | 2,024 | 1,266 | 1,531 | 2,346 | 2,060 | 1,346 |
| 2010 | 1,283 | 1,587 | 1,686 | 1,232 | 1,361 | 1,734 | 1,522 | 1,401 |
| 2011 | 1,299 | 1,893 | 1,479 | 1,423 | 1,139 | 1,841 | 1,855 | 1,053 |
| 2012 | 1,208 | 1,673 | 1,678 | 1,175 | 1,966 | 2,134 | 2,296 | 1,854 |
| 2013 | 1,146 | 1,755 | 1,738 | 1,138 | 1,442 | 1,821 | 2,369 | 1,318 |
| 2014 | 1,364 | 1,681 | 1,731 | 1,267 | 1,657 | 2,188 | 1,836 | 1,612 |
| 2015 | 1,631 | 2,031 | 1,885 | 1,542 | 1,795 | 1,882 | 2,166 | 1,606 |
| 2016 | 1,281 | 1,542 | 1,606 | 1,218 | 1,996 | 1,765 | 1,891 | 1,699 |
| 2017 | 1,487 | 2,013 | 1,620 | 1,299 | 2,097 | 1,414 | 2,370 | 1,855 |
| 2018 | 1,598 | 1,673 | 1,819 | 1,531 | 1,657 | 1,793 | 2,317 | 1,517 |
| 2019 | 1,223 | 1,790 | 1,532 | 1,157 | 1,702 | 1,536 | 2,033 | 1,526 |

We used regularizing Guassian priors centred on zero, with Normal(0, 0.1) for each *β*, and a Normal(0, 0.5) for *ω*. A plot of the joint distribution between income and wellbeing imposed by the priors alone confirmed there was no apparent slope or change point implied by their regularizing effect.

##### Figure S6. Prior predictive check


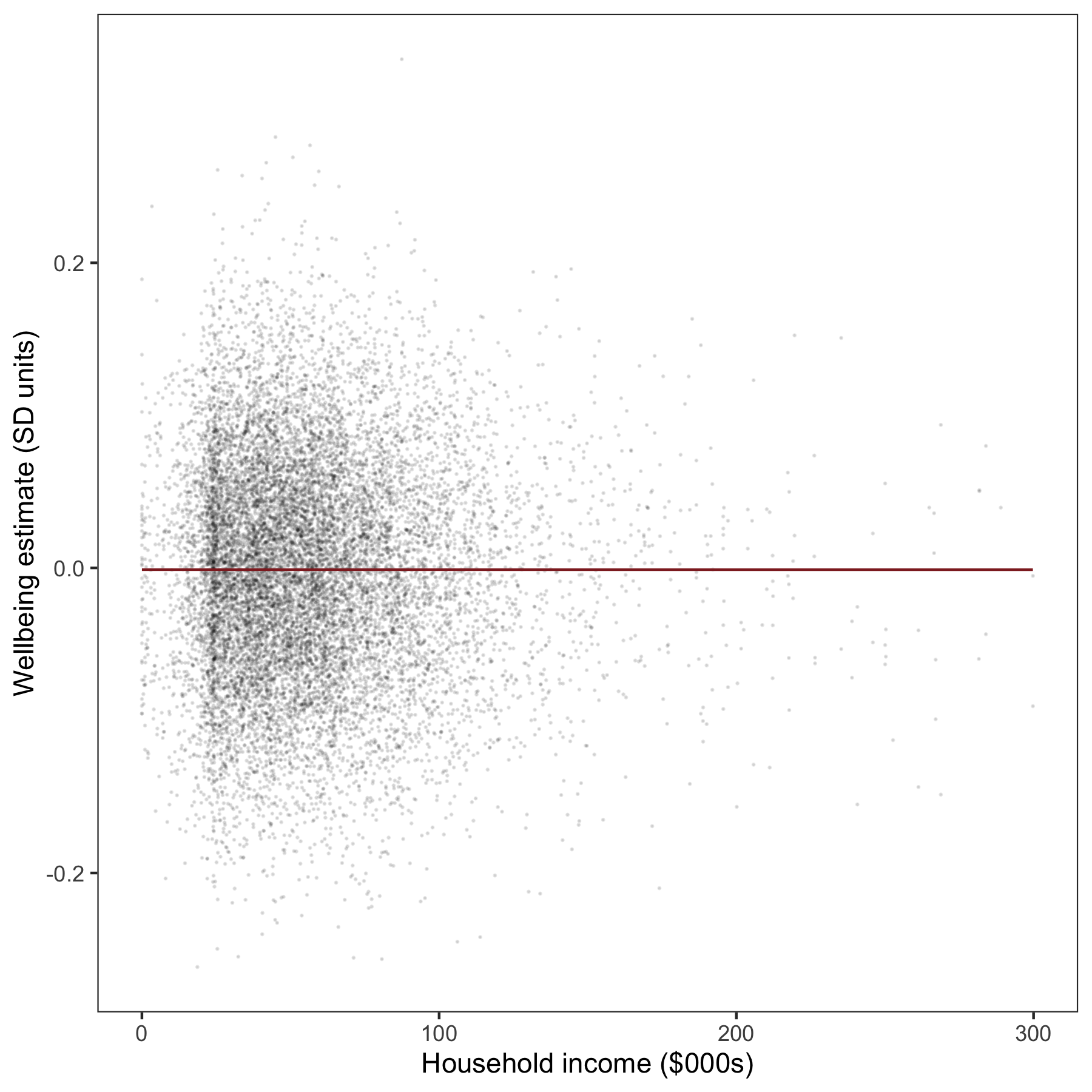


Figure legend: The joint distribution between wellbeing and income for a single year, imposed by the priors. The solid blue line indicates a smoothed average (loess).

Examination of the residual plots from the piecewise models of affective wellbeing confirms the residuals were evenly distributed around zero and there was little indication of heteroscedascity. Overall, there was little evidence that the piecewise models of affective wellbeing were not appropriate for the data.

##### Figure S7. Residual plots for piecewise affective wellbeing models


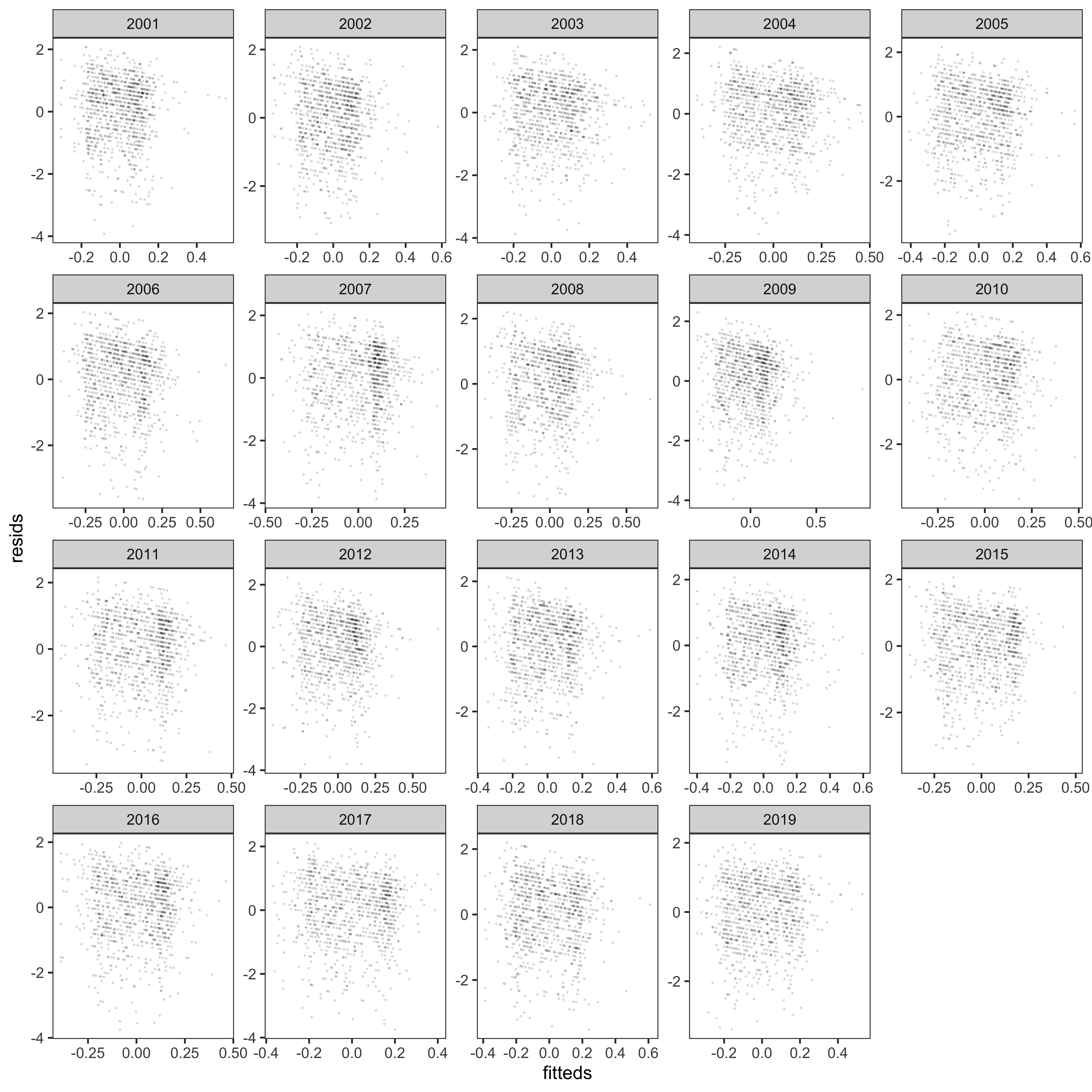


Figure legend: The joint distribution between residual and fitted values for each year.

#### Model predictions (2001-2019)

The posterior estimates of the piecewise model indicated that the relationship between affective wellbeing and income changed over time between 2001 and 2019. One implication of such a change is the disparity in affective wellbeing between income groups has increased. This will occur as more people fall below the change point over time and so are subject to the steep region of the function where income and affective wellbeing are strongly related. To directly examine the implications of our model for such inequities in affective wellbeing, we used the model to estimate or *predict* the affective wellbeing of two different income levels: one income level which was above the change point in 2001 but fell below it by 2019 ($50K/yr); and another level which always remained above the change points over the same period ($75K/yr). The figure below presents the affective wellbeing levels for each income level as well as the difference in affective wellbeing between them (∆).

##### Figure S8. Affective wellbeing at $50K/yr and $75K/yr from 2001-2019


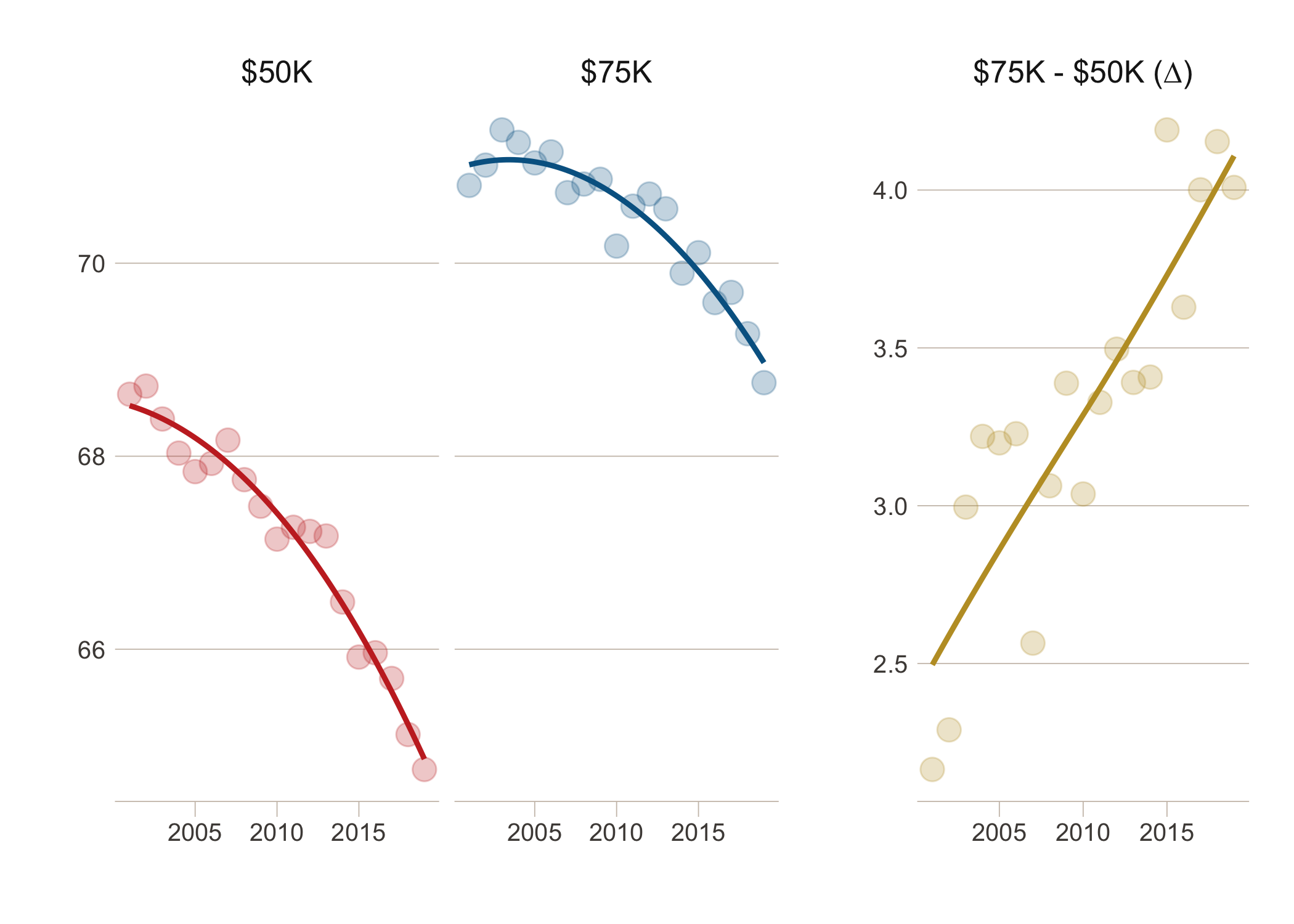


Figure legend: The difference (∆) in affective wellbeing between a household income of $50K per year and a household income of $75K per year has increased between 2001 and 2019.

The figure above shows the disparity in affective wellbeing between two fixed income levels, $50K/year (traversing the change points) and $75K/year (above the change points). In 2001, a household income of $50K/year achieved an above average level of affective wellbeing relative to everyone else that year, however by 2019 affective wellbeing had declined to lower than average levels relative to the population. By comparison, a household income of $75K/year enjoyed a higher level of affective wellbeing which decreased more slowly over the same period. The difference (∆) in affective wellbeing between these two income levels doubled over this period. The increasing disparity in affective wellbeing between the rich and the poor implies that affective wellbeing has became more inequitable over time.

## Appendix

#### Bayesian linear, log-linear and piecewise-linear models

The mean and standard deviation (±se) values of the posterior distributions for each coeficient from the piecewise-linear, log-linear and linear Bayesian models are shown in the table below.

##### Table S3. Bayesian coefficients

|  | Piecewise-linear (±se) | | | |  | Log-linear (±se) | |  | Linear (±se) | |
| --- | --- | --- | --- | --- | --- | --- | --- | --- | --- | --- |
| Year | β0 | β1 | β2 | ω |  | β0 | β1 |  | β0 | β1 |
| 2001 | 69.16 (0.052) | 0.37 (0.051) | 0.12 (0.026) | 43.57 (5.384) |  | 60.48 (0.62) | 0.3 (0.024) |  | 68.23 (0.155) | 0.2 (0.016) |
| 2002 | 69.27 (0.061) | 0.39 (0.059) | 0.12 (0.028) | 43.61 (6.336) |  | 59.79 (0.663) | 0.33 (0.026) |  | 68.32 (0.158) | 0.21 (0.016) |
| 2003 | 69.77 (0.074) | 0.37 (0.048) | 0.13 (0.033) | 49.87 (7.107) |  | 57.52 (0.722) | 0.42 (0.028) |  | 68.3 (0.165) | 0.23 (0.017) |
| 2004 | 69.57 (0.054) | 0.42 (0.045) | 0.14 (0.029) | 49.68 (4.487) |  | 56.67 (0.711) | 0.44 (0.027) |  | 68.06 (0.164) | 0.26 (0.017) |
| 2005 | 70.01 (0.061) | 0.45 (0.049) | 0.1 (0.032) | 53.84 (5.595) |  | 57.22 (0.704) | 0.41 (0.027) |  | 68.06 (0.162) | 0.24 (0.016) |
| 2006 | 70 (0.052) | 0.48 (0.05) | 0.12 (0.028) | 52.88 (4.598) |  | 56.17 (0.703) | 0.47 (0.027) |  | 68.29 (0.16) | 0.25 (0.016) |
| 2007 | 69.6 (0.041) | 0.6 (0.066) | 0.11 (0.024) | 47.75 (3.444) |  | 56.49 (0.72) | 0.45 (0.028) |  | 68.35 (0.163) | 0.24 (0.016) |
| 2008 | 69.62 (0.072) | 0.5 (0.068) | 0.13 (0.032) | 52.58 (7.853) |  | 57.03 (0.704) | 0.43 (0.027) |  | 68.38 (0.161) | 0.25 (0.017) |
| 2009 | 69.91 (0.057) | 0.44 (0.048) | 0.13 (0.028) | 57.84 (5.865) |  | 58.44 (0.673) | 0.38 (0.025) |  | 68.54 (0.161) | 0.25 (0.016) |
| 2010 | 69.42 (0.061) | 0.49 (0.066) | 0.1 (0.03) | 56.29 (6.938) |  | 57.76 (0.697) | 0.39 (0.026) |  | 67.94 (0.157) | 0.23 (0.016) |
| 2011 | 69.86 (0.044) | 0.53 (0.052) | 0.1 (0.025) | 56.19 (4.384) |  | 56.9 (0.598) | 0.43 (0.023) |  | 68.17 (0.142) | 0.24 (0.014) |
| 2012 | 69.82 (0.053) | 0.51 (0.054) | 0.13 (0.024) | 57.51 (5.608) |  | 57.96 (0.598) | 0.4 (0.023) |  | 68.37 (0.141) | 0.25 (0.014) |
| 2013 | 70.05 (0.047) | 0.41 (0.041) | 0.1 (0.026) | 63.96 (5.446) |  | 59.34 (0.621) | 0.34 (0.024) |  | 68.3 (0.141) | 0.23 (0.014) |
| 2014 | 69.12 (0.052) | 0.48 (0.051) | 0.12 (0.026) | 58.96 (5.654) |  | 56.65 (0.644) | 0.42 (0.025) |  | 67.63 (0.144) | 0.24 (0.014) |
| 2015 | 69.92 (0.048) | 0.42 (0.035) | 0.06 (0.03) | 70.8 (4.97) |  | 56.6 (0.633) | 0.41 (0.024) |  | 67.23 (0.142) | 0.24 (0.014) |
| 2016 | 69.06 (0.058) | 0.46 (0.049) | 0.09 (0.029) | 62.82 (6.476) |  | 56.51 (0.628) | 0.41 (0.024) |  | 67.15 (0.146) | 0.23 (0.014) |
| 2017 | 69.42 (0.046) | 0.44 (0.037) | 0.08 (0.027) | 68.94 (4.864) |  | 56.68 (0.62) | 0.4 (0.024) |  | 66.95 (0.141) | 0.23 (0.015) |
| 2018 | 68.9 (0.048) | 0.45 (0.037) | 0.1 (0.027) | 68.67 (4.954) |  | 55.86 (0.617) | 0.41 (0.024) |  | 66.51 (0.142) | 0.26 (0.014) |
| 2019 | 68.55 (0.069) | 0.39 (0.036) | 0.12 (0.032) | 73.46 (7.905) |  | 55.95 (0.611) | 0.4 (0.024) |  | 66.21 (0.146) | 0.25 (0.015) |

The table of model parameters for each year show that the $\beta_{1}$ slopes for piecewise-linear, log-linear and linear model for affective wellbeing and income do not show a sustained trend over the 19 year period. The $\beta_{1}$ coefficients increase in the initial 5-10 years and then decline. Despite this common pattern, the linear slope appears to show the most sustained increase while the log-linear slope shows a relatively less sustained increase. Overall, the pattern of changes among the models indicates the function between income and affective wellbeing has in fact become slightly steeper over the period.

#### OLS linear, log-linear and piecewise-linear models

The temporal trends in coefficients from OLS models were largely consistent with those observed in the Bayesian models above. The change point (in real income) increased between 2001 and 2019, however there were few other trends over time.

##### Table S4. MLE coefficients from piecewise-linear, linear, log-linear OLS models

|  | Piecewise-linear (±se) | | | |  | Log-linear (±se) | |  | Linear (±se) | |
| --- | --- | --- | --- | --- | --- | --- | --- | --- | --- | --- |
| Year | β0 | β1 | β2 | ω |  | β0 | β1 |  | β0 | β1 |
| 2001 | 62.17 (0.68) | 1.74 (0.26) | 0.45 (0.27) | 38.86 (4) |  | 64.32 (0.35) | 0.3 (0.02) |  | 64.9 (0.3) | 0.77 (0.06) |
| 2002 | 61.72 (0.75) | 1.88 (0.28) | 0.47 (0.3) | 39.19 (3.94) |  | 63.97 (0.38) | 0.33 (0.03) |  | 64.74 (0.32) | 0.82 (0.06) |
| 2003 | 61.73 (0.68) | 1.75 (0.23) | 0.57 (0.25) | 42.56 (4.5) |  | 62.74 (0.41) | 0.42 (0.03) |  | 64.24 (0.33) | 0.92 (0.06) |
| 2004 | 61.1 (0.63) | 1.76 (0.2) | 0.54 (0.23) | 48.46 (4.55) |  | 62.08 (0.42) | 0.44 (0.03) |  | 63.59 (0.33) | 0.98 (0.06) |
| 2005 | 61.47 (0.55) | 1.57 (0.15) | 0.32 (0.19) | 57.4 (4.53) |  | 62.31 (0.42) | 0.41 (0.03) |  | 64 (0.32) | 0.86 (0.06) |
| 2006 | 60.75 (0.64) | 1.81 (0.19) | 0.4 (0.21) | 51.35 (3.89) |  | 61.61 (0.43) | 0.47 (0.03) |  | 64.05 (0.32) | 0.86 (0.06) |
| 2007 | 57.38 (1.03) | 3.18 (0.4) | 0.43 (0.41) | 35.5 (2.19) |  | 61.65 (0.44) | 0.45 (0.03) |  | 64.42 (0.32) | 0.76 (0.05) |
| 2008 | 60.08 (0.77) | 2.03 (0.25) | 0.45 (0.26) | 44.57 (3.49) |  | 61.94 (0.44) | 0.43 (0.03) |  | 64.22 (0.32) | 0.79 (0.05) |
| 2009 | 61.27 (0.62) | 1.55 (0.16) | 0.41 (0.19) | 55.5 (4.54) |  | 62.66 (0.42) | 0.38 (0.02) |  | 64.22 (0.33) | 0.79 (0.05) |
| 2010 | 58.12 (0.82) | 2.62 (0.29) | 0.44 (0.3) | 39.38 (2.27) |  | 61.57 (0.38) | 0.43 (0.02) |  | 64.12 (0.28) | 0.74 (0.04) |
| 2011 | 60.4 (0.62) | 1.75 (0.17) | 0.41 (0.18) | 52.04 (3.48) |  | 62.2 (0.38) | 0.4 (0.02) |  | 64.16 (0.28) | 0.75 (0.04) |
| 2012 | 60.17 (0.62) | 1.6 (0.16) | 0.37 (0.18) | 54.92 (3.92) |  | 60.98 (0.41) | 0.42 (0.02) |  | 63.59 (0.29) | 0.71 (0.04) |
| 2013 | 60.38 (0.49) | 1.37 (0.11) | 0.15 (0.14) | 71.9 (4.36) |  | 60.81 (0.41) | 0.41 (0.02) |  | 63.11 (0.29) | 0.73 (0.04) |
| 2014 | 59.67 (0.49) | 1.36 (0.11) | 0.3 (0.14) | 68.47 (4.76) |  | 60.06 (0.4) | 0.41 (0.02) |  | 62.28 (0.28) | 0.75 (0.04) |
| 2015 | 60.11 (0.45) | 1.14 (0.09) | 0.28 (0.13) | 80.71 (6.53) |  | 59.85 (0.4) | 0.4 (0.02) |  | 61.88 (0.29) | 0.75 (0.04) |
| 2016 | 58.29 (0.99) | 2.62 (0.37) | 0.42 (0.38) | 37.15 (2.62) |  | 62.04 (0.42) | 0.38 (0.03) |  | 64.22 (0.31) | 0.68 (0.05) |
| 2017 | 59.54 (0.61) | 1.64 (0.16) | 0.33 (0.18) | 55.59 (3.63) |  | 60.73 (0.41) | 0.41 (0.02) |  | 63.15 (0.28) | 0.71 (0.04) |
| 2018 | 60.24 (0.48) | 1.35 (0.11) | 0.21 (0.14) | 69.03 (4.43) |  | 60.76 (0.39) | 0.4 (0.02) |  | 63.05 (0.28) | 0.7 (0.04) |
| 2019 | 62.01 (0.51) | 1.29 (0.12) | 0.29 (0.15) | 63.13 (4.88) |  | 62.97 (0.39) | 0.34 (0.02) |  | 64.57 (0.28) | 0.66 (0.04) |

**In-sample fit statistics**. Our primary objective was to understand the relationships between the parameters of our model, and so in-sample fit statistics such as R^2^ are irrelevant for this objective. Moreover, our use of regularized priors will bias such in-sample estimates towards zero as regularization deliberately sacrifices in-sample variance for out-of-sample accuracy, which obviously hinders interpretation of the in-sample values. Nevertheless, some readers may find it useful to compare the in-sample fit statistics between the three different model types employed here: piecewise, linear and log-linear, as well as between the conditional and unconditional models. Because the usual fit statistics such as R^2^ present a problem for Bayesian fits, as the variance of the predicted values can be larger than the variance of the data results in R^2^ values greater than 1, we present the in-sample statistics from OLS model fits (without regularization or penalized likelihoods).

##### Table S5. Adjusted R-squared for affective wellbeing and income

| Year | Linear | Linear (conditional) | Log-linear | Log-linear (conditional) | Piecewise | Piecewise (conditional) |
| --- | --- | --- | --- | --- | --- | --- |
| 2001 | 0.014 | 0.116 | 0.013 | 0.116 | 0.016 | 0.117 |
| 2002 | 0.016 | 0.119 | 0.015 | 0.119 | 0.018 | 0.121 |
| 2003 | 0.019 | 0.117 | 0.020 | 0.118 | 0.021 | 0.118 |
| 2004 | 0.023 | 0.140 | 0.024 | 0.141 | 0.026 | 0.142 |
| 2005 | 0.021 | 0.134 | 0.022 | 0.134 | 0.025 | 0.136 |
| 2006 | 0.023 | 0.130 | 0.026 | 0.132 | 0.028 | 0.132 |
| 2007 | 0.020 | 0.131 | 0.026 | 0.134 | 0.028 | 0.136 |
| 2008 | 0.022 | 0.137 | 0.025 | 0.138 | 0.027 | 0.139 |
| 2009 | 0.022 | 0.142 | 0.023 | 0.143 | 0.026 | 0.144 |
| 2010 | 0.021 | 0.125 | 0.025 | 0.128 | 0.027 | 0.130 |
| 2011 | 0.023 | 0.123 | 0.022 | 0.124 | 0.027 | 0.126 |
| 2012 | 0.019 | 0.134 | 0.021 | 0.135 | 0.023 | 0.135 |
| 2013 | 0.019 | 0.139 | 0.020 | 0.140 | 0.024 | 0.142 |
| 2014 | 0.021 | 0.145 | 0.021 | 0.146 | 0.025 | 0.148 |
| 2015 | 0.020 | 0.145 | 0.019 | 0.146 | 0.023 | 0.147 |
| 2016 | 0.018 | 0.132 | 0.021 | 0.134 | 0.024 | 0.136 |
| 2017 | 0.018 | 0.145 | 0.019 | 0.146 | 0.023 | 0.147 |
| 2018 | 0.018 | 0.140 | 0.019 | 0.141 | 0.023 | 0.143 |
| 2019 | 0.017 | 0.132 | 0.016 | 0.132 | 0.021 | 0.134 |
| Overall | 0.018 | 0.132 | 0.019 | 0.133 | 0.022 | 0.134 |

A comparison of the relative differences in adjusted R^2^ values between models indicates the piecewise model produced a slightly higher in-sample fit over the other two models, while the linear model tended to have the lowest adjusted R^2^. The log-linear fit varied between both over the 19 years. As expected, adding covariates increased the adjusted R-squared in each case, but it did not change the relative difference between models.

Three things need to be observed when interpreting the R^2^ values: 1) While the R^2^ between income and affective wellbeing is low (~2% and ~14% in the unconditional and conditional models, respectively), the effect over the wider population will aggregate to be much larger; 2) affective wellbeing (and to a lesser extent, income) is an imperfect and noisy measure so any effect of income on happiness is likely to be larger than that measured here; 3) Finally, affective wellbeing is measured on a subjective and arbitrary Likert scale so a small change in affective wellbeing at different points in the scale may represent a large change in some other functional/real-world outcome (e.g., risk of suicide). This is all to say that quantifying the real-world impact of small changes in variation reported here is difficult and speculative without further investigation.

## References

Bürkner, P.-C., 2017. brms: An R package for Bayesian multilevel models using Stan. Journal of Statistical Software 80, 1–28. <https://doi.org/10.18637/jss.v080.i01>

Stan Development Team, 2019. RStan: The R interface to Stan.
